# Diagnostic Utility of Ki‐67 Index on Cell Block Material for Grading Medullary Thyroid Carcinoma

**DOI:** 10.1002/dc.70102

**Published:** 2026-02-16

**Authors:** Tieying Hou, Hector Mesa, Bianca Puello, Brent Molden

**Affiliations:** ^1^ Department of Pathology and Laboratory Medicine Indiana University School of Medicine Indianapolis Indiana USA; ^2^ Department of Pathology University of Maryland School of Medicine Baltimore Maryland USA; ^3^ AmeriPath Inc. Indianapolis Indiana USA

**Keywords:** cell block, grading, Ki‐67 proliferation index, medullary thyroid carcinoma

## Abstract

**Introduction:**

An international grading system for medullary thyroid carcinoma (IMTCGS) was established in 2022. High‐grade MTC is defined by the presence of one or more of the following features: mitotic index ≥ 5 per 2 mm^2^, Ki‐67 proliferative index ≥ 5%, or tumor necrosis. Fine‐needle aspiration (FNA) is the primary diagnostic tool for MTC; however, tumor grading in cell blocks remains underexplored.

**Materials and Methods:**

A total of 60 FNA cases diagnosed as MTC or metastatic MTC between 2000 and 2025 were identified. Of these, 15 met the inclusion criteria: cell blocks with > 500 tumor cells and corresponding surgical specimens available. The Ki‐67 proliferation index was assessed in hotspot regions using an online platform.

**Results:**

The study included 11 adults and 4 pediatric/adolescents with a median age of 50 years (range: 8–76). Based on the IMTCGS grading criteria, six cases were classified as high‐grade and nine as low‐grade. In low‐grade tumors, the Ki‐67 proliferation index on surgical resections ranged from < 1% to 4%, and all corresponding cell blocks showed Ki‐67 rates of ≤ 1%. High‐grade tumors exhibited Ki‐67 indices ranging from 7% to 24%, with mitotic figures ranging from 1 to 14 per 2 mm^2^. Among these, two cell blocks showed Ki‐67 rates of 5%, while the remaining four had rates between < 1% and 2%. Four of six patients with high‐grade MTC developed distant metastases to the liver, lung, or bone, and one died of disease 35 months after diagnosis. None of the low‐grade tumors developed distant metastasis.

**Conclusions:**

Grading MTC using the Ki‐67 proliferation index on cell blocks is challenging because of low cellularity and intratumoral heterogeneity in Ki‐67 expression. In our study, this approach tended to undergrade high‐grade MTC.

## 1 Introduction

Medullary thyroid carcinoma (MTC) is a rare endocrine tumor of the thyroid arising from calcitonin‐secreting C cells. Recent studies have highlighted the prognostic significance of proliferative activity and tumor necrosis. In 2020, a three‐tiered system and a two‐tiered system were proposed [1, 2]. Subsequently, an international MTC grading system (IMTCGS) was validated in 2022 [3], defining high‐grade MTC as tumors exhibiting at least one of the following features: mitotic index ≥ 5 per 2 mm^2^, Ki‐67 proliferation index ≥ 5%, or tumor necrosis. Since its establishment, multiple studies have confirmed that the IMTCGS is an independent and strong prognostic predictor, demonstrating strong association with disease‐specific survival [4, 5], overall survival, or distant metastasis‐free survival [6].

Most MTC are initially detected by fine‐needle aspiration (FNA). Although cell‐block preparations are typically adequate for diagnostic ancillary testing (e.g., calcitonin), tumor grading in cell blocks remains underexplored. Viswanathan et al. [7] reported that the Ki‐67 proliferation index assessed in cell‐block material demonstrated 92% concordance with corresponding surgical specimens. The specificity of Ki‐67 on FNA for identifying high‐grade MTC was 100%, but the sensitivity was only 38%.

In this study, we correlated the mitotic activity, necrosis, and Ki‐67 proliferation index in cell blocks and resection specimens of 15 cases of MTC to further evaluate the accuracy of grading in cytology samples.

## 2 Materials and Methods

After institutional review board approval, all FNA cases diagnosed as either primary or metastatic MTC between January 2000 and September 2025 were retrieved from the pathology database. Cases were included if the cell block had a cellularity exceeding 500 cells and a corresponding surgical specimen available within our system. Clinical and radiologic data were retrieved from electronic medical records. Both cytology and surgical resection slides were retrospectively reviewed by TH.

Direct smears from FNA specimens were stained using both Diff‐Quik (DQ) and Papanicolaou (PAP) stains. For cell block preparation, cell pellets obtained after centrifugation were fixed in CytoRich Red medium. HistoGel was added to the pellets, and the solidified cell pellet cone was placed inside a biopsy bag for routine tissue processing. Following paraffin embedding, 4 μm‐thick sections were cut and stained with hematoxylin and eosin (H&E).

Immunohistochemical staining for Ki‐67 was performed on both cell block and surgical resection specimens using the Ki‐67 antibody (MIB‐1, sc‐10,861, Santa Cruz Biotechnology) on a Dako automated platform. Because Ki‐67 immunostaining has not been validated on direct smears in our laboratory, this method was not pursued. The Ki‐67 proliferation index was quantified using the online tool: DEEPLIIF (deepliif.org). For each case, two to three images of representative hot spots with at least 500 cells were captured and uploaded to the platform. The final Ki‐67 rate was the average of the values obtained from all submitted pictures. All statistical analyses were performed using R (version 4.x). Fisher's exact test was used to evaluate associations between high‐grade tumors and categorical variables, including sex, margin status, distant metastasis, lymphovascular invasion, and MEN syndrome. The Mann–Whitney U test was applied to continuous variables, such as tumor size and age. Statistical significance was determined at an alpha level of 0.05.

## 3 Results

**FIGURE 1:**
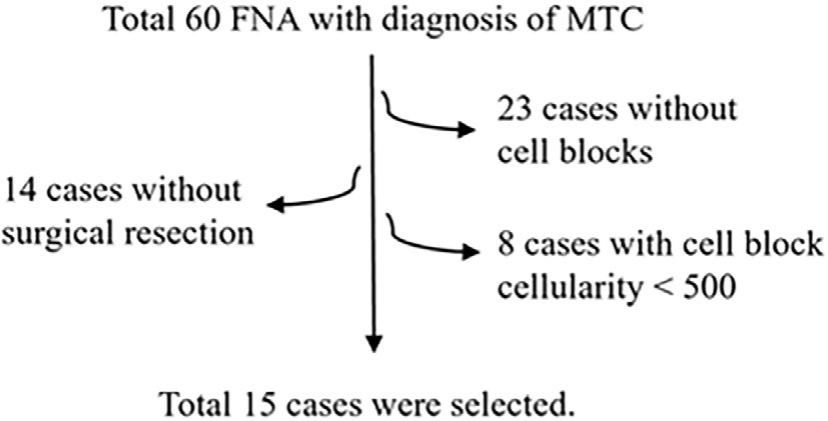
Workflow for case selection.

Between January 2000 and September 2025, a total of 60 FNA cases were diagnosed as primary or metastatic MTC. Of these, 23 cases lacked cell block material; 8 had cell blocks with cellularity below 500 cells, and 14 did not have corresponding surgical specimens. The remaining 15 cases were adequate for evaluation (Figure 1).

The patient cohort included nine females and six males, with a median age of 50 (range: 8–76 years) (Table 1). Eleven patients were adults, while four were pediatric/adolescent cases. Tumor size ranged from 0.4 cm to 8.5 cm in greatest dimension. Four patients had germ line *RET* gene mutations including three with Multiple Endocrine Neoplasia 2A (MEN 2A) and 1 with MEN 2B. All patients with MEN syndrome presented at a young age (range: 8–34 years) and had bilateral disease. Based on histologic features and Ki‐67 proliferation index, nine cases were classified as low‐grade MTC and six as high‐grade on the surgical resection. Among the high‐grade cases, four had metastasis involving the lung, liver, or bone. One patient died of the disease after 35 months of follow up. None of the low‐grade tumors developed distant metastasis. No statistically significant associations were detected between clinical variables or pathologic features and high‐grade tumors (Supplemental Table 1), likely due to the small cohort size; however, a trend toward a higher rate of distant metastasis was observed in high‐grade MTC.

**TABLE 1:** Clinical and pathologic information of all cases.

Patients	sex	age	Tumor size (cm)	Bilateral	Margin	LVI	Staging	Grading	Distant metastasis	MEN syndrome	prognosis	Follow up time (m)
1	19	M	0.8	Yes	Negative	Yes	mpT1aN1b	Low	No	MEN 2A	Alive	220
2	18	F	0.4	Yes	Negative	No	mpT1aN0	Low	No	MEN 2A	Alive	80
3	54	F	3.5	No	Negative	Yes	pT2N1b	Low	No	Unknown	Alive	23
4	50	M	8.5	No	Negative	No	pT3aN0	Low	No	Unknown	Alive	15
5	76	M	0.5	Yes	Negative	No	mpT1aN0	Low	No	Negative	Alive	68
6	34	M	1.5	Yes	Negative	Yes	mpT1bN1a	Low	No	MEN 2A	Alive	11
7	66	M	2.4	No	Negative	Yes	pT2N0	Low	No	Negative	Alive	53
8	27	F	2.8	No	Positive	Yes	pT3bN1b	Low	No	Negative	Alive	43
9	30	F	N/A	No	Negative	N/A	pTXN1b^§^	Low	No	Somatic*	Alive	36
10	64	F	2.1	No	Negative	Yes	pT2N1b	High	No	Negative	Alive	43
11	76	M	N/A	Yes	N/A¶	Yes	mpT4aN1bM1	High	Yes, liver	Unknown	Dead	35
12	8	F	N/A	Yes	N/A¶	N/A	mpT2N1bM1	High	Yes, lung	MEN 2B	Alive	34
13	13	F	2.5	No	Negative	Yes	pT2N0	High	No	Negative	Alive	49
14	57	F	4.5	No	Positive	Yes	pT3aN1bM1	High	Yes, bone	Somatic*	Alive	84
15	53	F	3	No	Positive	Yes	pT2N1bM1	High	Yes, liver	Unknown	Alive	10

* Somatic *RET* M918T mutation detected; N/A: not available.

^§^ No primary tumor was identified in the total thyroidectomy specimen. Somatic *RET* M918T mutation was identified in tumor cells.

^¶^ These two patients did not receive total thyroidectomy due to multiple comorbidities and extensive diseases. Tumor size and staging were determined based on the clinical and radiologic findings.

**FIGURE 2:**
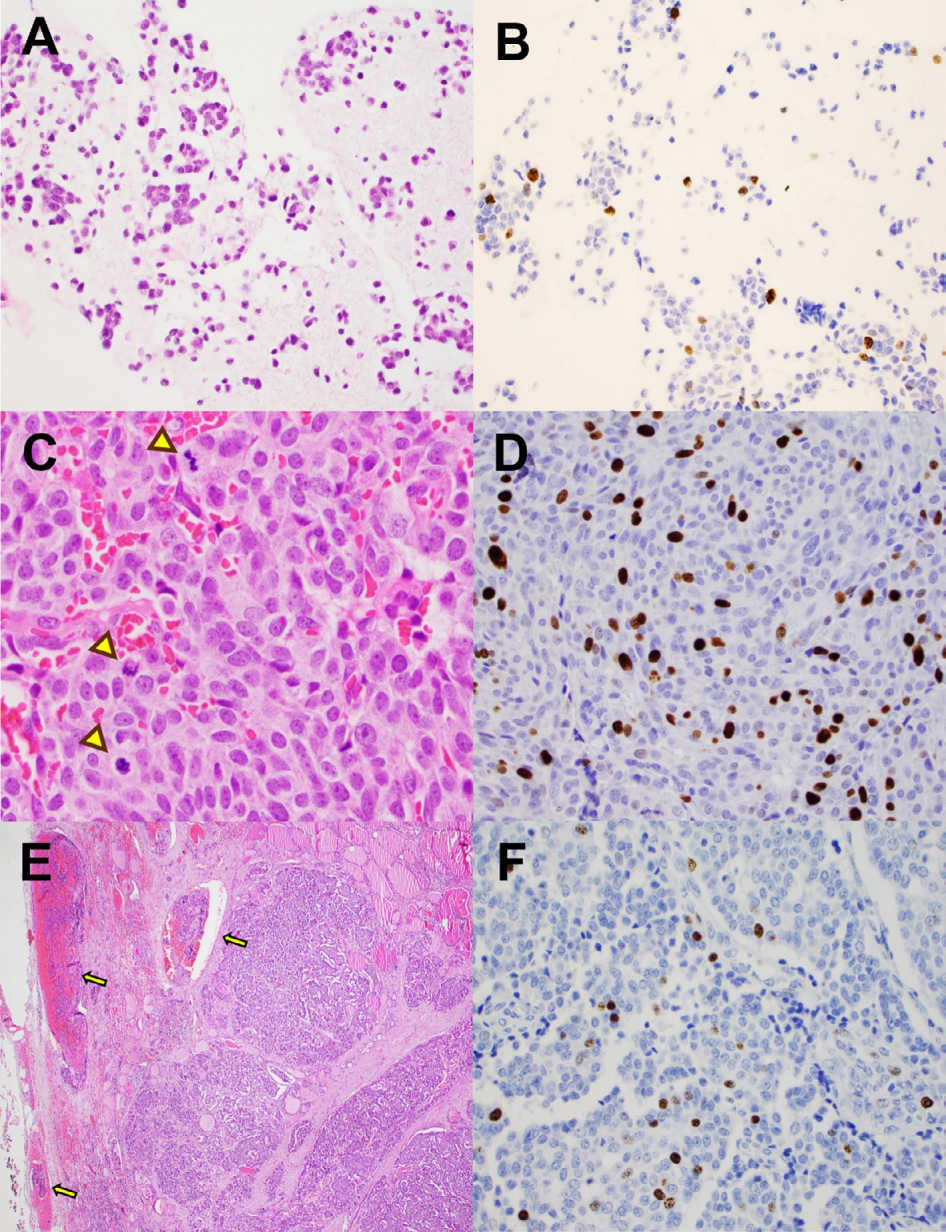
FNA specimens and surgical resection from patient 14. A: Cell block from thyroid FNA containing > 500 tumor cells. B: Ki‐67 rate on the cell block was 5%. C: Lateral neck lymph node dissection showed metastatic MTC with increased mitotic activity (arrowhead). D: Ki‐67 proliferation index in the metastatic lymph node was 13%. E: Total thyroidectomy specimen from the same patient obtained 4 years earlier demonstrated extensive lymphovascular invasion (arrow). F: Ki‐67 proliferation rate in the thyroidectomy was up to 8%. No significantly increased mitotic activity was observed. [Color figure can be viewed at wileyonlinelibrary.com]

FNA specimens were obtained from the thyroid (10 cases) or cervical lymph nodes (5 cases) (Table 2). Of the five cervical lymph node FNAs, three were performed before thyroidectomy for initial diagnosis or staging (patients 3, 9, and 11), and two were obtained from neck lymph node recurrences (patients 1 and 14). The primary thyroidectomy specimen for patient 1, performed in the 1980s, was unavailable for review. Patient 14 underwent thyroidectomy 4 years prior to recurrence; this specimen showed high‐grade MTC with a Ki‐67 proliferation index of 8% (Figure 2). Corresponding surgical specimens were collected from the same anatomic site as the FNA in all cases except for patient 12, whose FNA was from thyroid, while the surgical specimen was a lung wedge resection. The interval between FNA and surgical resections ranged from < 1 to 3 months, except for patient 14, who underwent neck dissection 22 months after the FNA diagnosis.

**TABLE 2:** Correlation between FNA specimens and surgical resection.

	FNA				Interval	Surgical resection				
	Location	Mitosis	Necrosis	Ki67	Months	Location	Mitosis	Necrosis	Ki67	Grade
1	Neck LN	0	no	< 1%	1	Neck LN	0	no	< 1%	low
2	Thyroid	0	no	< 1%	1	Thyroid	1	no	1%	low
3	Neck LN	0	no	< 1%	1.5	Neck LN	1	no	1%	low
4	Thyroid	0	no	1%	1	Thyroid	0	no	1%	low
5	Thyroid	0	no	1%	2.5	Thyroid	0	no	1%	low
6	Thyroid	0	no	1%	1.5	Thyroid	0	no	2%	low
7	Thyroid	0	no	1%	1	Thyroid	2	no	3%	low
8	Thyroid	0	no	1%	1.5	Thyroid	1	no	3%	low
9	Neck LN	0	no	1%	1	Neck LN	1	no	4%	low
10	Thyroid	2	no	2%	2.5	Thyroid	3	no	7%	high
11	Neck LN	0	no	< 1%	1	Neck LN	4	yes	8%	high
12	Thyroid	0	no	5%	1	Lung	1	no	9%	high
13	Thyroid	0	no	2%	2	Thyroid	2	no	9%	high
14	Neck LN	0	no	5%	22	Neck LN	9	no	13%	high
15	Thyroid	0	no	< 1%	1	Thyroid	14	no	24%	high

**FIGURE 3:**
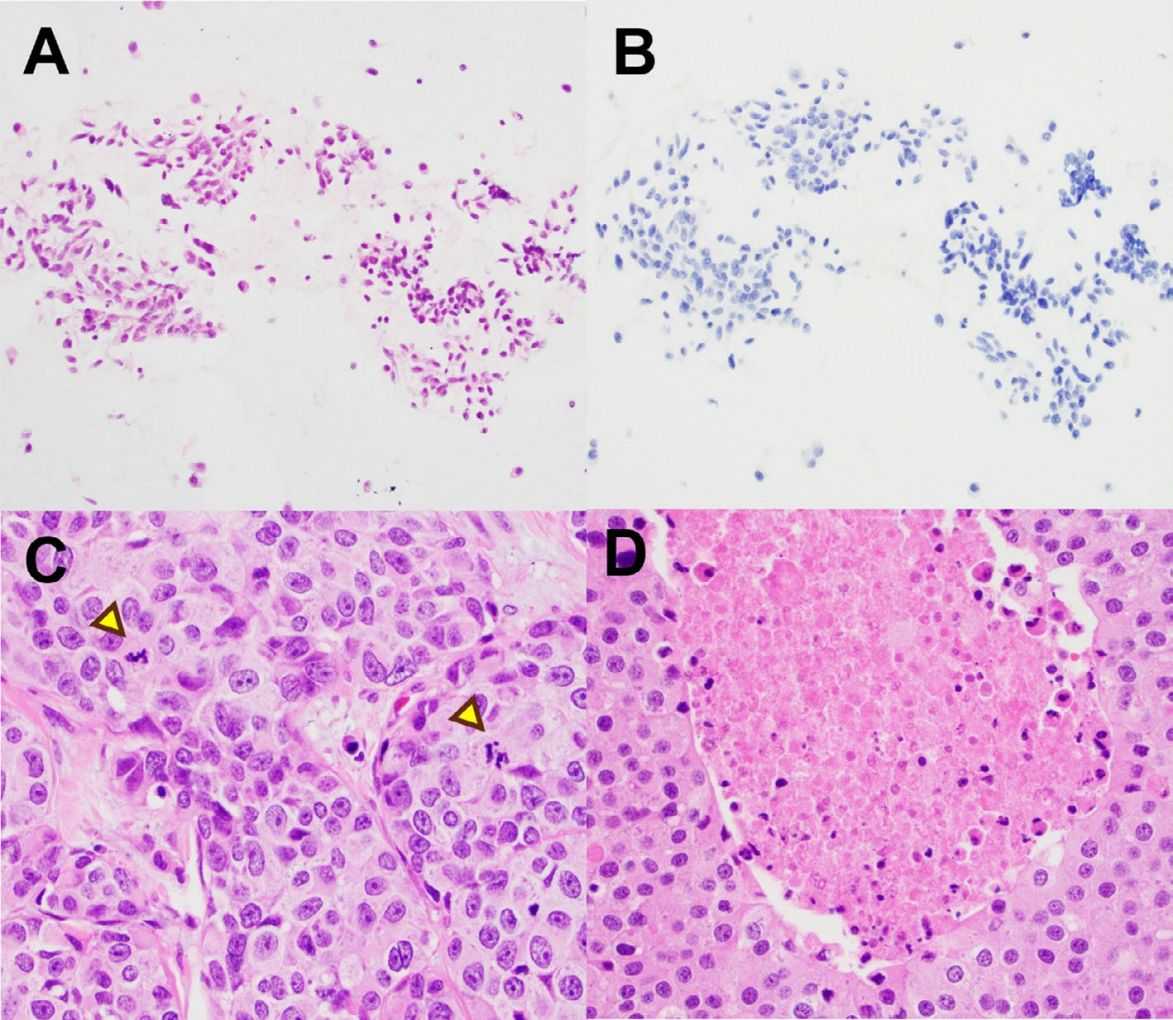
FNA and surgical resection from neck lymph node in patient 11. A‐B: Cell block was cellular (A) with Ki67 proliferation rate < 1% (B). C‐D: Neck dissection demonstrated metastatic MTC with mitotic activity (arrowhead in C) and focal necrosis (D). [Color figure can be viewed at wileyonlinelibrary.com]

**FIGURE 4:**
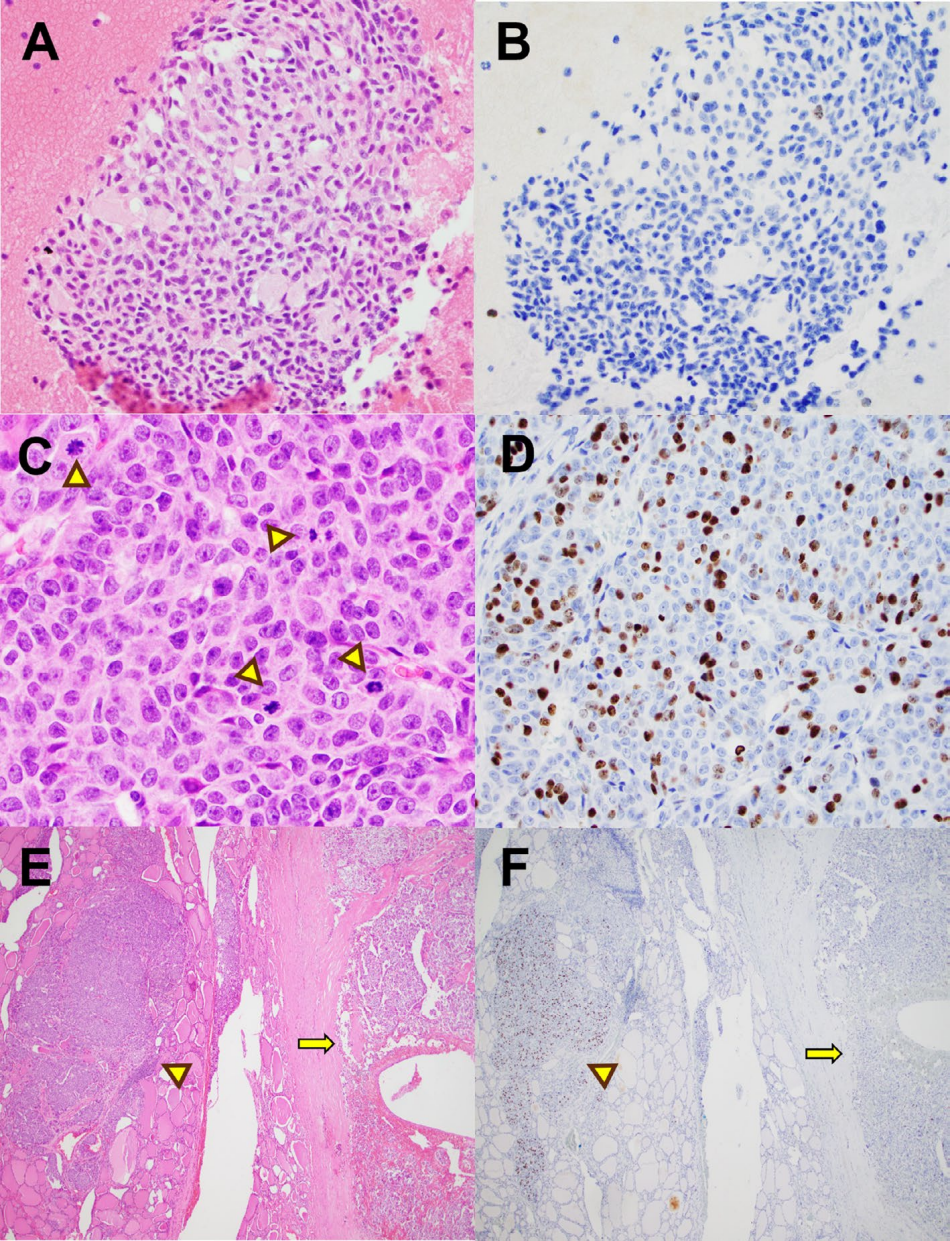
FNA biopsy and total thyroidectomy from patient 15. A‐B: Cell block from thyroid FNA containing > 500 tumor cells (A) with Ki‐67 rate < 1% (B). C‐D: H&E section showed MTC with increased mitotic activity (arrowhead in C). Ki‐67 proliferation rate was up to 24% (D). E‐F: At low magnification, H&E section revealed heterogeneous Ki‐67 distribution: a hot spot with high proliferation rate (24%) on the left (arrowhead in E and F) and low index (3%) on the right (arrow in E and F). [Color figure can be viewed at wileyonlinelibrary.com]

In resection specimens, all low‐grade MTC had Ki‐67 proliferation indices ranging from < 1% to 4%, and mitotic counts between 0 and 2 per 2 mm^2^. The corresponding FNA cell blocks also demonstrated low proliferation rates of ≤ 1%. In resection specimens, the six high‐grade tumors exhibited Ki‐67 indices ranging from 7% to 24% and mitotic rates from 1 to 14 per 2 mm^2^. Tumor necrosis was observed in only one surgical specimen (Figure 3). Among the corresponding FNA cell blocks, two cases showed Ki‐67 rates of 5% (patient 12 and 14), while the remaining four had proliferation indices between < 1% and 2% (Figures 2, 3, 4). One case (patient 10) demonstrated a mitotic count of two per 2 mm^2^. No mitotic figures or necrosis were identified in the other cell blocks or direct smears. Notably, Ki‐67 distribution on surgical resections varied widely across different areas; for example, in patient 15 it ranged from 3% to 24% (Figure 4). The overall concordance rate between cell‐block–based grading and surgical resection was 73.3% (11 of 15 cases). However, the concordance rate for high‐grade tumors was only 33.3% (2 of 6 cases). Ki‐67 on cell blocks demonstrated high sensitivity (100%) but low specificity (33.3%) for identifying low‐grade tumors. Conversely, it showed high specificity (100%) but low sensitivity (33.3%) for detecting high‐grade tumors (Supplemental Table 2).

## 4 Discussion

After its validation in 2022 [3], the IMTCGS two‐tiered grading system has been established as a potent prognostic predictor in MTC. High‐grade MTCs are associated with decreased overall, disease‐specific, distant‐metastasis‐free, and locoregional‐recurrence‐free survival [1, 2, 3, 5, 8, 9, 10].

The grading system incorporates three parameters: mitotic rate, Ki‐67 proliferation index, and tumor necrosis. Both mitotic activity and Ki‐67 index are assessed in proliferative hotspots [3]. In our study, 6 of the 15 cases (40%) were classified as high‐grade MTC and all of them had a Ki‐67 index > 5%, but only two had a mitotic activity > 5 per 2 mm^2^, and only one exhibited tumor necrosis. This discrepancy between Ki‐67 labeling index and mitotic activity has also been noted in prior studies [3, 11]. Consistent with previous observations, Ki‐67 served as the primary determinant of high‐grade status in our cohort [11].

For low‐grade MTC cases, cytology‐based grading was fully concordant with surgical specimens. In contrast, only two of six high‐grade cases were correctly identified as high‐grade on cytology. Although the overall concordance between cell‐block‐based grading and surgical specimens was 73.3%, this was largely driven by the perfect agreement in low‐grade cases, suggesting that cell blocks tend to undergrade high‐grade MTC. These results are aligned with Viswanathan et al. [7] who reported Ki‐67 ≥ 5% on FNA shows low sensitivity but high specificity for predicting high‐grade MTC. The clinical implications of this limitation require further study. Currently, the extent of surgery for MTC is guided by clinicoradiologic findings and serum calcitonin levels [12]. Total thyroidectomy with routine central compartment lymph node dissection is recommended for most patients, even in the absence of clinically evident lymphadenopathy, while lateral neck dissection is reserved for those with radiologic or clinical evidence of metastasis. Although histologic grading is a strong prognostic factor, it currently plays a limited role in guiding initial surgical management and is primarily used for postoperative risk stratification and surveillance planning.

Similar challenges have been described in the cytologic grading of pancreatic neuroendocrine neoplasms, where Ki‐67 assessment tends to undergrade tumors—particularly WHO grade 2 lesions [13, 14, 15]. The sensitivity and specificity for detecting grade 1 and grade 3 tumors are typically higher than for grade 2 tumors [15]. Limited cellularity in cell blocks contributes to this discrepancy. Abi‐Raad, Rita et al. [13] reported that when cell blocks contain more than 1000 tumor cells, the correlation of the Ki‐67 index between cell blocks and surgical specimens improved substantially for grading pancreatic neuroendocrine neoplasms. However, achieving highly cellular thyroid FNA cell blocks is challenging due to the organ's vascularity and the risk of procedure‐induced hematoma, which could compromise the airway.

Sampling error due to intratumoral Ki‐67 heterogeneity is another important factor. Heterogeneous Ki‐67 expression has been reported in both primary gastroenteropancreatic neuroendocrine neoplasms and synchronous or metachronous liver metastases [16, 17, 18, 19]. In the study by Yang et al. [19], 47% of cases demonstrated intratumoral heterogeneity in the Ki‐67 index, resulting in discrepant tumor grades. In our study, case 15 showed a wide Ki‐67 range (3%–24%) in the resection specimen, whereas the corresponding cell block showed a Ki‐67 index of < 1%, consistent with sampling variability.

The final potential contributing factor is that the cytology fixation medium may influence Ki‐67 antibody immunoreactivity. The MIB‐1 clone is commonly used for Ki‐67 immunostaining. Buonocore et al. [20] reported that, among 10 evaluated cell blocks, the mean MIB‐1 labeling index in CytoLyt fixed specimens was significantly lower than in formalin fixed specimens (10% vs. 47%). In addition, the positive percentage in the CytoLyt fixed group was reduced by an average of 37% compared with their formalin fixed counterparts. They also found that an alternative Ki‐67 clone, 30–9 (Ventana), demonstrated markedly superior reactivity relative to MIB‐1 in CytoLyt fixed samples. Based on these results, the authors recommended use of the Ki‐67 30–9 antibody for CytoLyt fixed cell blocks. Additionally, an earlier study by Gong et al. [21] observed that Ki‐67 performance in formalin fixed cell blocks was superior to that in CytoLyt fixed ThinPrep specimens.

Clinical factors such as older age, male sex, and elevated serum calcitonin levels have been associated with poorer prognosis of MTC [3, 12]. In addition to tumor grade, several pathologic features have been reported as independent prognostic factors, including higher pathologic stage, positive margin status, postoperative serum CEA and calcitonin levels, and the presence of vascular invasion [3]. In this small cohort, patients with high‐grade MTC showed a higher risk of distant metastasis (4 of 6 cases, 67%) compared with those with low‐grade tumors (0 of 9 cases). Other clinical variables did not reach statistical significance, primarily due to the limited sample size. In the study by Prete et al. [9], a significant difference in locoregional‐recurrence‐free survival between high‐ and low‐grade tumors was observed only among patients with localized disease, suggesting that grading may have the greatest prognostic utility in early‐stage MTC. The relationship between MTC grade and *RET* mutation status also remains uncertain. While Censi et al. [4] reported that patients with *RET* mutations were more frequently classified as high‐grade, other studies have found no significant difference in *RET* mutation status between low‐ and high‐grade tumors [3].

Ki‐67 levels can vary during disease progression, and metastatic tumors may exhibit higher proliferative activity [17, 18, 22, 23]. Consistent with this, patient 14 showed an increase in Ki‐67 index from the primary thyroidectomy specimen (8%) to the recurrent cervical lymph node metastasis (13%). Because of such variability, we initially applied strict inclusion criteria, requiring an interval of less than 6 months between FNA and surgical resection and, ideally, specimen collection from the same anatomical site. However, very few cases met these requirements in addition to the other inclusion criteria. Given the small cohort size, we elected to include patient 14 and patient 12 despite deviations from the predefined criteria. Patient 14 underwent neck dissection 22 months after FNA, and in patient 12, the FNA specimen was obtained from a neck lymph node, whereas the surgical specimen came from a lung wedge. Therefore, findings from these two patients should be interpreted with caution. Future multicenter collaboration and expanded cohort sizes will be valuable to further investigate this topic.

## 5 Conclusions

Identifying high‐grade MTC using cytology materials remains challenging, largely because of limited cellularity in cell blocks and intratumoral heterogeneity in proliferative activity. In the future, if cytology‐based grading is shown to influence upfront clinical management, obtaining more cellular blocks or performing a concurrent core biopsy may improve grading accuracy.

## Author Contributions

Design, data analysis, manuscript draft, table, and figures: T.H., H.M.; manuscript editing: B.P., B.M.

## Funding

The authors have nothing to report.

## Conflicts of Interest

The authors declare no conflicts of interest.

## Supporting information


**Supplemental Table 1:** Statistical Associations with High‐grade Tumors.
Although no statistically significant associations were observed between clinical or pathologic features and high‐grade tumors—possibly due to the small sample size—a trend toward a higher incidence of distant metastasis was noted in high‐grade MTC.
**Supplemental Table 2:** Performance Characteristics of Ki‐67 in Cell Block.
Ki‐67 assessment on cell block material showed excellent sensitivity (100%) but limited specificity (33.3%) for low‐grade tumors, whereas the opposite pattern was observed for high‐grade tumors, with perfect specificity (100%) but low sensitivity (33.3%).

## Data Availability

The data that support the findings of this study are available on request from the corresponding author. The data are not publicly available due to privacy or ethical restrictions.

## References

[1] B. Alzumaili , B. Xu , P. M. Spanheimer , et al., “Grading of Medullary Thyroid Carcinoma on the Basis of Tumor Necrosis and High Mitotic Rate Is an Independent Predictor of Poor Outcome,” Modern Pathology 33, no. 9 (2020): 1690–1701, 10.1038/s41379-020-0532-1. 32313184 PMC7483270

[2] T. L. Fuchs , A. J. Nassour , A. Glover , et al., “A Proposed Grading Scheme for Medullary Thyroid Carcinoma Based on Proliferative Activity (Ki‐67 and Mitotic Count) and Coagulative Necrosis,” American Journal of Surgical Pathology 44, no. 10 (2020): 1419–1428, 10.1097/PAS.0000000000001505. 32452872 PMC7641183

[3] B. Xu , T. L. Fuchs , S. Ahmadi , et al., “International Medullary Thyroid Carcinoma Grading System: A Validated Grading System for Medullary Thyroid Carcinoma,” Journal of Clinical Oncology 40, no. 1 (2022): 96–104, 10.1200/JCO.21.01329. 34731032 PMC8683221

[4] S. Censi , F. Galuppini , C. Clausi , et al., “Tumor Grade and Molecular Characteristics Associated With Survival in Sporadic Medullary Thyroid Carcinoma,” Thyroid 34, no. 2 (2024): 177–185, 10.1089/thy.2023.0482. 38047536

[5] J. Ni , X. Zhang , Y. Liu , and Y. Ling , “A Comparison of the Predictive Value of International Medullary Thyroid Carcinoma Grading System (IMTCGS) With That of Other Risk Factors in a Chinese Medullary Thyroid Carcinoma Cohort,” Clinical Endocrinology 102, no. 5 (2025): 589–599, 10.1111/cen.15195. 39749465

[6] V. Rai , A. Saha , S. Mehta , et al., “International Medullary Thyroid Carcinoma Grading System: An Indian Tertiary Care Centre Experience,” European Archives of Oto‐Rhino‐Laryngology 281, no. 3 (2024): 1571–1579, 10.1007/s00405-023-08341-x. 38010402

[7] K. Viswanathan , D. B. Behrman , and D. J. Lubin , “Grading Medullary Thyroid Carcinoma on Fine‐Needle Aspiration Cytology Specimens With the International Medullary Thyroid Carcinoma Grading System: A Cytologic‐Histologic Correlation,” Cancer Cytopathology 132, no. 4 (2024): 224–232, 10.1002/cncy.22778. 38062948

[8] D. J. Lubin , D. B. Behrman , S. Goyal , et al., “Independent Validation of the International Grading System for Medullary Thyroid Carcinoma: A Single Institution Experience,” Modern Pathology 36, no. 9 (2023): 100235, 10.1016/j.modpat.2023.100235. 37270155 PMC10528047

[9] A. Prete , L. Torregrossa , C. Gambale , et al., “The Usefulness of the International Grading System in the Management of Sporadic Medullary Thyroid Carcinoma,” Thyroid 35, no. 4 (2025): 387–396, 10.1089/thy.2024.0444. 39930944

[10] E. Vissio , F. Maletta , J. Fissore , et al., “External Validation of Three Available Grading Systems for Medullary Thyroid Carcinoma in a Single Institution Cohort,” Endocrine Pathology 33, no. 3 (2022): 359–370, 10.1007/s12022-022-09719-z. 35583706

[11] F. Torricelli , G. Santandrea , C. Botti , et al., “Medullary Thyroid Carcinomas Classified According to the International Medullary Carcinoma Grading System and a Surveillance, Epidemiology, and End Results‐Based Metastatic Risk Score: A Correlation With Genetic Profile and Angioinvasion,” Modern Pathology 36, no. 9 (2023): 100244, 10.1016/j.modpat.2023.100244. 37307881

[12] S. A. Wells, Jr. , S. L. Asa , H. Dralle , et al., “Revised American Thyroid Association Guidelines for the Management of Medullary Thyroid Carcinoma,” Thyroid 25, no. 6 (2015): 567–610, 10.1089/thy.2014.0335. 25810047 PMC4490627

[13] R. Abi‐Raad , J. P. Lavik , A. L. Barbieri , X. Zhang , A. J. Adeniran , and G. Cai , “Grading Pancreatic Neuroendocrine Tumors by Ki‐67 Index Evaluated on Fine‐Needle Aspiration Cell Block Material,” American Journal of Clinical Pathology 153, no. 1 (2020): 74–81, 10.1093/ajcp/aqz110. 31415691

[14] A. Appelstrand , F. Bergstedt , A. K. Elf , H. Fagman , and P. Hedenstrom , “Endoscopic Ultrasound‐Guided Side‐Fenestrated Needle Biopsy Sampling Is Sensitive for Pancreatic Neuroendocrine Tumors but Inadequate for Tumor Grading: A Prospective Study,” Scientific Reports 12, no. 1 (2022): 5971, 10.1038/s41598-022-09923-1. 35396490 PMC8993931

[15] J. S. Pyo , N. Y. Kim , K. W. Min , I. H. Oh , D. H. Lim , and B. K. Son , “Diagnostic Accuracy of Ki‐67 Labeling Index in Endoscopic Ultrasonography‐Fine‐Needle Aspiration Cytology and Biopsy of Pancreatic Neuroendocrine Neoplasms,” Diagnostics (Basel) 13, no. 17 (2023): 2756–2766, 10.3390/diagnostics13172756. 37685294 PMC10487187

[16] A. Couvelard , L. Deschamps , P. Ravaud , et al., “Heterogeneity of Tumor Prognostic Markers: A Reproducibility Study Applied to Liver Metastases of Pancreatic Endocrine Tumors,” Modern Pathology 22, no. 2 (2009): 273–281, 10.1038/modpathol.2008.177. 18997736

[17] F. Grillo , M. Albertelli , M. P. Brisigotti , et al., “Grade Increases in Gastroenteropancreatic Neuroendocrine Tumor Metastases Compared to the Primary Tumor,” Neuroendocrinology 103, no. 5 (2016): 452–459, 10.1159/000439434. 26337010

[18] C. Shi , R. S. Gonzalez , Z. Zhao , et al., “Liver Metastases of Small Intestine Neuroendocrine Tumors: Ki‐67 Heterogeneity and World Health Organization Grade Discordance With Primary Tumors,” American Journal of Clinical Pathology 143, no. 3 (2015): 398–404, 10.1309/AJCPQ55SKOCYFZHN. 25696798 PMC4354931

[19] Z. Yang , L. H. Tang , and D. S. Klimstra , “Effect of Tumor Heterogeneity on the Assessment of Ki67 Labeling Index in Well‐Differentiated Neuroendocrine Tumors Metastatic to the Liver: Implications for Prognostic Stratification,” American Journal of Surgical Pathology 35, no. 6 (2011): 853–860, 10.1097/PAS.0b013e31821a0696. 21566513

[20] D. J. Buonocore , F. Konno , A. A. Jungbluth , et al., “CytoLyt Fixation Significantly Inhibits MIB1 Immunoreactivity Whereas Alternative Ki‐67 Clone 30‐9 Is Not Susceptible to the Inhibition: Critical Diagnostic Implications,” Cancer Cytopathology 127, no. 10 (2019): 643–649, 10.1002/cncy.22170. 31398281 PMC8375359

[21] Y. Gong , X. Sun , C. W. Michael , S. Attal , B. A. Williamson , and C. W. Bedrossian , “Immunocytochemistry of Serous Effusion Specimens: A Comparison of ThinPrep vs Cell Block,” Diagnostic Cytopathology 28, no. 1 (2003): 1–5, 10.1002/dc.10219. 12508174

[22] K. J. Keck , A. Choi , J. E. Maxwell , et al., “Increased Grade in Neuroendocrine Tumor Metastases Negatively Impacts Survival,” Annals of Surgical Oncology 24, no. 8 (2017): 2206–2212, 10.1245/s10434-017-5899-y. 28560597 PMC5772651

[23] Y. Zen and N. Heaton , “Elevated Ki‐67 Labeling Index in 'synchronous Liver Metastases' of Well Differentiated Enteropancreatic Neuroendocrine Tumor,” Pathology International 63, no. 11 (2013): 532–538, 10.1111/pin.12108. 24274715

