# Supplementary material for: Diagnostic Utility of Ki‐67 Index on Cell Block Material for Grading Medullary Thyroid Carcinoma
Source: Diagn Cytopathol. 2026 Feb 16;54(5):380–7. doi: 10.1002/dc.70102 (PMC13047298; doi:10.1002/dc.70102)
Supplement: Supplementary file 1 — Supplemental Table 1: Statistical Associations with High‐grade Tumors. Although no statistically significant associations were observed between clinical or pathologic features and high‐grade tumors—possibly due to the small sample size—a trend toward a higher incidence of distant metastasis was noted in high‐grade MTC. Supplemental Table 2: Performance Characteristics of Ki‐67 in Cell Block. Ki‐67 assessment on cell block material showed excellent sensitivity (100%) but limited specificity (33.3%) for low‐grade tumors, whereas the opposite pattern was observed for high‐grade tumors, with perfect specificity (100%) but low sensitivity (33.3%). [file DC-54-380-s001.docx]

| **Supplemental Table 1. Statistical Associations with High-grade tumors** | | |
| --- | --- | --- |
| Variable | Test | p-value |
| Sex | Fisher’s exact test | 0.64 |
| Margin Status |  | 0.18 |
| Distant Metastasis |  | 0.07 |
| Lymphovascular Invasion |  | 0.29 |
| MEN Syndrome |  | 0.55 |
| Tumor Size | Mann–Whitney U test | 0.41 |
| Age |  | 0.22 |

| **Supplemental Table 2. Performance Characteristics of Ki-67 in cell block** | | | | | |
| --- | --- | --- | --- | --- | --- |
| **Grade** | **TP** | **FN** | **FP** | **TN** | **Total** |
| **Low** | 9 | 0 | 4 | 2 | 15 |
| **High** | 2 | 4 | 0 | 9 | 15 |
| **Total** | 11 | 4 | 4 | 11 | 30 |

| **Metric** | **Low-grade %** | **High-grade %** |
| --- | --- | --- |
| **Sensitivity** | 100 | 33.3 |
| **Specificity** | 33.3 | 100 |
| **Positive Predictive Value** | 69.2 | 100 |
| **Negative Predictive Value** | 100 | 69.2 |

TP: true positive; FN: false negative; FP: false positive; TN: true negative
